# Erosive potential of children’s chewable vitamin supplements: An in vitro investigation

**DOI:** 10.34172/joddd.41791

**Published:** 2024-12-14

**Authors:** Wannee Lertsooksawat, Yanisa Tanjaruphan, Thanawat Chaima, Thanawat Lerdpibulchai, Tanawan Wittayathawornwong, Chayada Teanchai, Rudee Surarit, Sivaporn Horsophonphong

**Affiliations:** ^1^Department of Pharmacology, Faculty of Dentistry, Mahidol University, Thailand; ^2^Undergraduate Program, Faculty of Dentistry, Mahidol University, Thailand; ^3^Department of Oral Biology, Faculty of Dentistry, Mahidol University, Thailand; ^4^Faculty of Dentistry, Siam University, Thailand; ^5^Research Office, Faculty of Dentistry, Mahidol University, Thailand; ^6^Department of Pediatric Dentistry, Faculty of Dentistry, Mahidol University, Thailand

**Keywords:** Children, Dental erosion, Dietary supplements, Vitamins

## Abstract

**Background.:**

Chewable vitamins are dietary supplements in which vitamins are combined with a flavor that appeals to children. The acidic nature of some vitamins and some flavoring agents may cause dental erosion. We investigated the effect of children’s chewable vitamin supplements on the loss of minerals in teeth.

**Methods.:**

Forty-two human tooth enamel samples were prepared and randomly assigned to seven groups (n=6). Six groups contained different chewable vitamin tablets dissolved in deionized water, and a control group contained only deionized water. Each tablet was ground before its dissolution, and its pH was measured. Each tooth sample was immersed in a vitamin solution and artificial saliva in periodic cycles for 14 days. Each sample was weighed before and after immersion to calculate its weight loss percentage. Scanning electron microscopy was used to investigate the morphology of the enamel surfaces. For statistical analysis, the weight loss percentages between groups were compared using Welch’s ANOVA, followed by post hoc Dunnett’s T3 analysis (*P*<0.05).

**Results.:**

All the tested supplements were acidic, with a pH range of 2.99‒4.77. Most of the vitamin groups tested led to significant weight loss; it was greater for the vitamin C groups than the multivitamin groups. Scanning electron microscopy revealed erosion and destruction of enamel surfaces following vitamin exposure.

**Conclusion.:**

All the chewable vitamin supplements were acidic, with the majority potentially eroding enamel. The erosive potential was less pronounced in vitamin supplements containing minerals.

## Introduction

 Dental erosion is the dissolution and loss of the minerals in tooth enamel due to contact with acids. It is a chemical process that does not involve plaque bacteria.^1^ Dental erosion can cause an irreversible loss of tooth structure, resulting in changes in the tooth’s vertical dimension, a poor tooth appearance, tooth hypersensitivity, pulpitis, and, ultimately, permanent tooth loss.^1,2^ The consumption of acidic foods and beverages is the leading cause of dental erosion.^3^

 People have become increasingly aware of their health and wellness in recent years. Physical activity and a healthy diet are two important aspects of good health, and efforts to stay healthy have included dietary supplements, which are considered nutritional products that boost the immune system and promote overall health.^4^ Dietary supplements are consumed by 29.5% to 34.6% of children, with mineral and vitamin supplements being the most popular. Of the supplements containing a single vitamin, vitamin C supplements are the most popular among children aged 2‒11 years.^5^ Chewable vitamin supplements are typically marketed for children because they contain nutritional elements and flavoring agents that appeal to children and motivate them.

 Although vitamin supplements are promoted as nutritional products that provide health benefits, some are acidic^6^ and may, therefore, erode tooth structure. Several studies have reported that functional drinks and beverages containing vitamins are mostly acidic and can cause dental erosion.^7,8^ Very few case studies are available that indicate chewing vitamin tablets erode teeth.^9,10^ No research has been done on the potentially erosive effects of chewable vitamin supplements, which, as they are crushed and chewed, directly contact the surfaces of teeth.Therefore, we aimed to investigate the effects of chewable vitamin supplements on the dissolution and loss of tooth minerals.

## Methods

 The experiment used sound human premolars as study samples. The tooth samples were tested with six commercially available chewable vitamin supplements for children, three of which contained only vitamin C, and three contained a multivitamin. Deionized water alone was used as a seventh control group. The name of each supplement, its manufacturer, and its major ingredients are presented in Table S1 (Supplementary file 1).

###  Sample size calculation 

 The seven tested groups consisted of three solutions containing a chewable vitamin C tablet, three solutions containing a chewable multivitamin, and one control solution of deionized water alone. The sample size was calculated using G*Power 3.120 with α = 0.05, β = 0.2, and a *Z*-value of 1.9599, and the means and standard deviations (SDs) were based on previous reports from Surarit et al.^8^ This yielded a total of 42 tooth samples (n = 6 per group).

###  Inclusion criteria

 The teeth selected for this study were caries-free human premolars with no decalcification, restoration, or macroscopic defects. The teeth were extracted for orthodontic reasons.

###  Preparation of tooth samples

 The extracted teeth were disinfected with 5% NaOCl for 30 minutes and stored in a 0.1% thymol solution. The samples were sectioned at the buccal and lingual enamel walls using a low-speed saw operating under a water coolant and polished with sandpaper to create tooth samples that were 4 × 4 × 1.5 mm in size, as measured by a Vernier caliper. The teeth were stored in deionized water until used for the experiment.

###  Preparation of chewable vitamin supplement solutions 

 A tablet of each vitamin supplement was ground and mixed with 3 mL of deionized water using a vortex mixer (Scientific Industries Inc, Bohemia, NY, USA) (to create a homogeneous chewable vitamin solution for the experiment.

###  Estimation of pH values of supplement solutions 

 The pH of each vitamin supplement solution was measured by a pH meter (Orion, Houston, TX, USA). Three solutions of each vitamin product were prepared, and the pH of the solutions was measured in triplicate to calculate the mean pH value.

###  Erosive cycles and evaluation of each sample’s weight loss

 In this study, enamel erosion was analyzed using the gravimetric method, a technique for evaluating dental erosion that measures a sample’s weight loss during an erosive process.^11^ Forty-two tooth samples were assigned randomly to seven groups, with deionized water used for a control group. Before the beginning of the immersion cycles, each tooth sample was dried in a desiccator for 24 hours, and the initial weight of each sample was measured using an analytical balance (Mettler-Toledo, Greifensee, Switzerland) with an accuracy of 0.01 mg. The erosive cycles lasted for 9 hours: each tooth sample was immersed in one of the test solutions for 1 hour, then in artificial saliva for 7 hours, and finally in one of the test solutions for 1 hour. Following that, the samples were stored overnight in artificial saliva. The artificial saliva composed of 0.7-mM CaCl_2_, 0.2-mM MgCl_2_, 4.0-mM KH_2_PO_4_, 30.0-mM KCl, and 20.0-mM HEPES, a formulation adopted from a study by ten Cate and Duijsters.^12^

**Figure 1:**
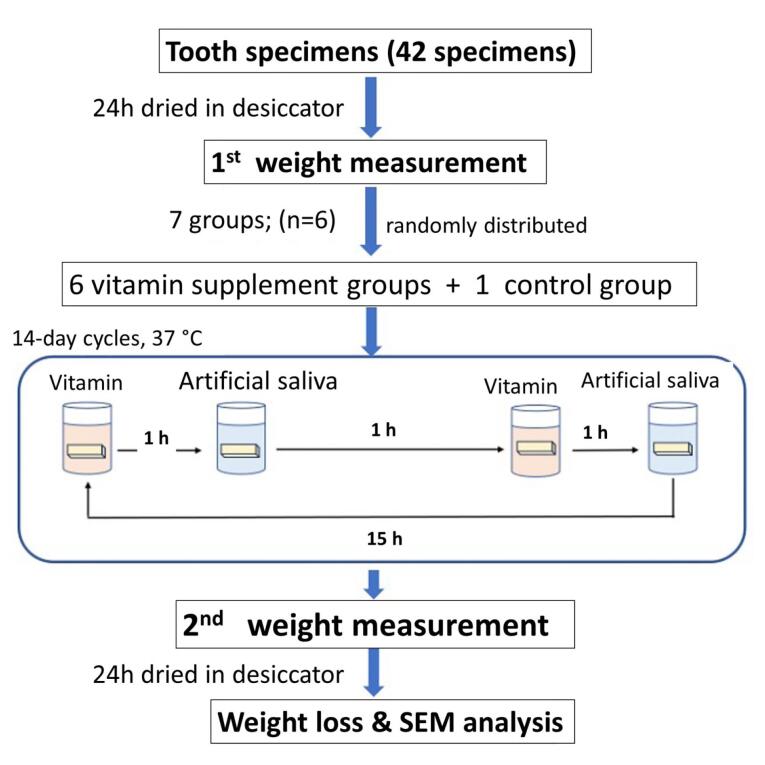
Diagram of the experiment’s workflow

 The cycles were repeated for 14 days. After that, the tooth samples were washed with deionized water and dried in a desiccator for 24 hours, and the final weight of each sample was measured. The weight loss percentages were calculated and reported as means and SDs. Figure 1 presents the experiment’s workflow.

###  Scanning electron microscopic findings

 After the 14-day immersion cycles, the samples were rinsed with deionized water and cleaned in an ultrasonic cleaner, dried, and sputter-coated with gold before being observed under a scanning electron microscope (SEM; JSM-6610LV; JEOL Ltd., Tokyo, Japan) at × 500, × 1000, and × 2000 magnifications using aperture diameters of 5 and 10 μm and an acceleration voltage of 15 kV.

###  Statistical analysis

 The means and SDs were reported for each sample’s weight loss, as was the pH of the vitamin supplement, including that of the control group. Statistical analysis was performed using SPSS 26.0 (IBM, Armonk, NY, USA). Welch’s ANOVA test, followed by post hoc Dunnett’s T3 test, was used to compare the weight loss percentages between groups; a *P* value of < 0.05 was considered a significant difference. Moreover, Pearson’s correlation coefficient was used to investigate the relationship between the pH value and weight loss percentage.

## Results

**Figure 2:**
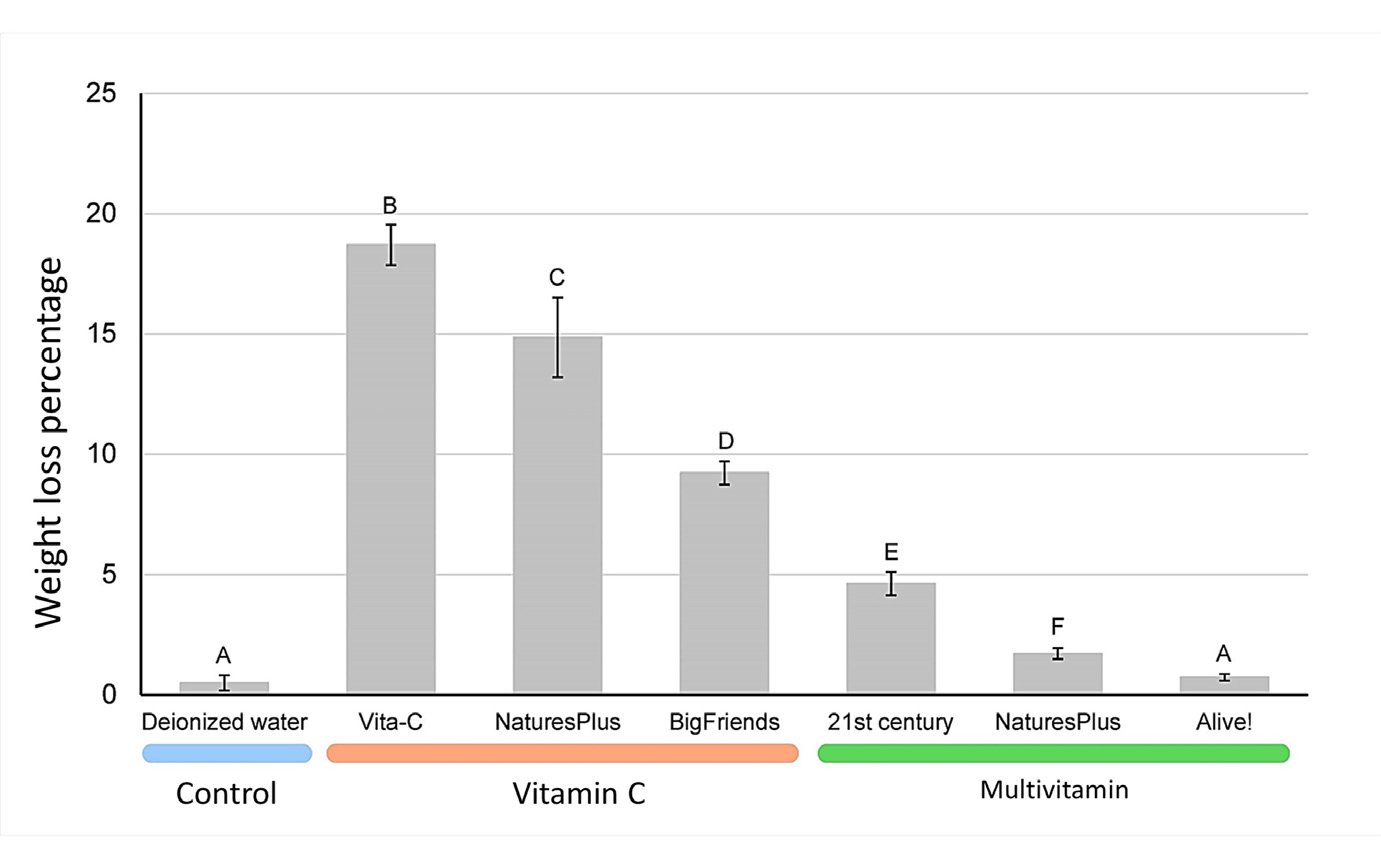
Weight loss percentages of tooth samples after 14 days of immersion. The different letters above the bars indicate statistically significant differences (*P* < 0.05)

 The pH values of the chewable vitamin supplements ranged from 2.99 to 4.77, with an average of 3.97. The Vita-C chewable vitamin C supplement had the lowest pH value and the Alive!Kids chewable multivitamin supplements had the highest pH value. Table 1 presents the pH of each vitamin supplement and the control group. Prior to tooth immersion, no significant differences in weight were observed across all the samples (*P* = 0.4244). Table 2 presents the weights of the samples before and after the 14-day immersion cycles. Figure 2 shows the weight loss percentages of tooth samples after the 14-day immersion cycles. The vitamin supplement that caused the most weight loss was Vita-C, followed by NaturesPlus vitamin C, BigFriends vitamin C, 21st Century multivitamin, NaturesPlusmultivitamin, and Alive! Kids multivitamin. All led to a significant difference in weight loss from the control group (*P* < 0.05), except the Alive! Kids multivitamin. Furthermore, the weight loss percentage for each vitamin group significantly differed from that for the other groups (*P* < 0.05), as shown in Figure 2.

**Table 1:** pH values of children’s chewable vitamin supplements and control

**Groups and products**	** Manufacturers**	**Mean pH value of the product (SD)**
Deionized water (control)		6.12 (0.266)
Vitamin C		
Vita-C orange flavor	Heaven Herb Co., Ltd., Pathum Thani, Thailand	2.99 (0.050)
NaturesPlus Vitamin C Children’s Chewable Supplement	Natural Organic, Inc. New York, USA	3.30 (0.094)
BigFriends Chewable Vitamin C	Natural Factors Canada., Monroe, USA	4.18 (0.026)
Multivitamin		
21st Century Children’s Multivitamin Supplement	21st Century HealthCare, Inc. Arizona, USA	4.77 (0.030)
NaturesPlus Multivitamin Children’s Chewable Supplement	Natural Organic, Inc. New York, USA	3.96 (0.075)
Alive!Kids Chewable Multivitamin	Nature’s Way Brands, LLC, Wisconsin, USA	4.62 (0.204)

**Table 2:** Weight of specimens before and after being immersed in the test solutions

**Groups and products**	**Weight of the samples (g)**		
	**Day 0: Before experiment (SD)**	**Day 14: After experiment (SD)**	**Weight loss** ** (SD)**
Deionized water (Control)	0.05210 (0.000808)	0.05184 (0.000829)	0.00026 (0.00017)
Vitamin C			
Vita-C orange flavor	0.05142 (0.001262)	0.04181 (0.001269)	0.00961 (0.00040)
NaturesPlusVitamin C Children’s Chewable Supplement	0.05264 (0.000497)	0.04482 (0.001022)	0.00782 (0.00087)
BigFriends Chewable Vitamin C	0.05205 (0.001023)	0.04725 (0.000828)	0.00481 (0.00031)
Multivitamin			
21st Century Children’s Multivitamin Supplement	0.05238 (0.000874)	0.04996 (0.000877)	0.00242 (0.00025)
NaturesPlus Multivitamin Children’s Chewable Supplement	0.05198 (0.001159)	0.05109 (0.001222)	0.00089 (0.00010)
Alive! Kids Chewable Multivitamin	0.05253 (0.001109)	0.05215 (0.001151)	0.00039 (0.00007)

 The pH value and weight loss percentage correlation revealed an inverse relationship (*r* = ‒0.806, *P* = 0.029). Moreover, a greater correlation was observed when the supplements that contained calcium (i.e., the NaturesPlus multivitamin and Alive! Kids multivitamin) were excluded (*r* = ‒0.975, *P* = 0.005).

**Figure 3:**
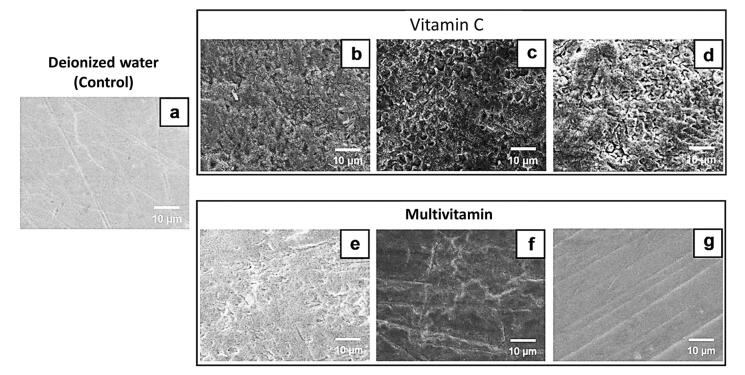
SEM images at × 1000 magnification of the enamel surface of samples after immersion in a vitamin supplement solution for a 14-day cycle: (a) deionized water; (b) Vita-C; (c) NaturesPlusvitamin C; (d) BigFriends vitamin C; (e) 21st Century multivitamin; (f) NaturesPlus multivitamin; (g) Alive! Kids multivitamin

**Figure 4:**
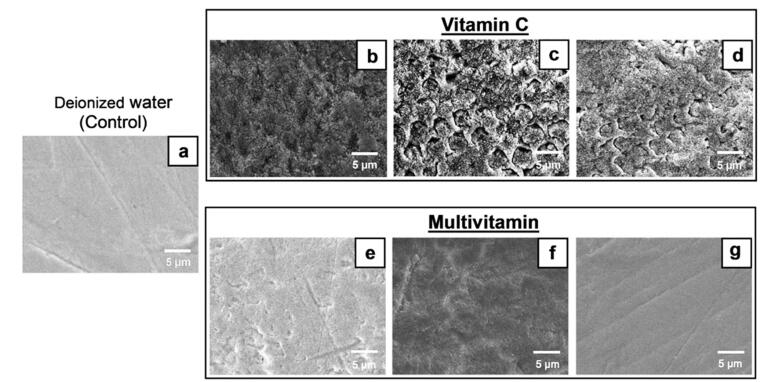
SEM images at × 2000 magnification of the enamel surface of the samples after immersion in a vitamin supplement solution for a 14-day cycle: (a) deionized water; (b) Vita-C; (c) NaturesPlus vitamin C; (d) BigFriends vitamin C; (e) 21st Century multivitamin; (f) NaturesPlus multivitamin; (g) Alive! Kids multivitamin

 After the 14-day immersion cycles, the tooth samples were viewed with SEM; Figures 3 and 4 show the micrographs. SEM analysis showed that samples in the control group had a consistently smooth surface with no apparent evidence of corrosion. However, some had scratches caused by sandpapering during the sample preparation process. A similar pattern was observed in a sample immersed in the Alive! Kids multivitamin solution, which was seen as a minimal alteration on the enamel’s surface. This was in contrast to samples immersed in other vitamin supplement solutions, in which destruction of enamel surfaces was found. Samples immersed in the vitamin C supplement solutions were severely corroded, and more enamel rods were seen to be exposed or destroyed compared with samples immersed in the multivitamin supplement solutions.

## Discussion

 This study investigated the effects of children’s chewable vitamin supplements, specifically those containing vitamin C alone or a multivitamin complex, on the dissolution and loss of tooth enamel. The results revealed that all the tested vitamins were acidic, with pH values ranging from 2.99 to 4.77; these values were lower than the critical pH value of 5.5 for enamel.^13^ This was consistent with previous research studies, which found various types of vitamin supplements to be acidic. Functional drinks containing vitamins, including vitamin waters, were found to have lower pH levels than the critical pH value for enamel.^7,8,14^ Furthermore, vitamin effervescent tablets were found to have an acidic pH ranging from 3.82 to 4.14.^15^ The acidity of chewable vitamin supplements could be due to the nature of vitamin C or ascorbic acid, the natural flavoring agents (e.g., apple, lemon, and pineapple), and the synthetic flavoring agents (e.g., citric acid and malic acid), which were added to make the supplements taste good.^16-18^

 This investigation used the gravimetric method, one of the standard techniques for evaluating erosion. It measures a sample’s weight loss to indicate the dissolution and loss of tooth enamel and evaluates enamel erosion.^11,19^

 The results indicated that, except Alive! Kids chewable multivitamins, all the tested chewable vitamin supplements for children had the potential to cause significant tooth enamel loss. Vita-C had the lowest pH (2.99), resulting in the most weight loss. The NaturesPlus vitamin C (pH = 3.30), BigFriends vitamin C (pH = 4.18), and 21st Centurymultivitamin (pH = 4.77) had a weight loss ranking that correlated with their pH values. On the other hand, the NaturesPlus multivitamin and Alive! Kids multivitamin resulted in weight losses that did not correspond to their pH values. This could be because both vitamin supplements contain minerals such as calcium, iron, and magnesium, which aid in remineralization or reduce demineralization.^15,20^ Our findings were consistent with those of Wegehaupt et al, who found that all the tested vitamin effervescent tablets were erosive, whereas some effervescent tablets containing both vitamins and minerals had no erosive effects.^15^

 The association between pH value and the erosive potential of chewable vitamin supplements was confirmed by Pearson’s correlation coefficient, which revealed an inverse relationship between pH value and a sample’s weight loss (*r* = ‒0.806). Additionally, when multivitamin supplements containing calcium were excluded, a greater correlation (*r* = ‒0.975) was observed, indicating a strong relationship between the pH of chewable vitamin supplements and their potential to cause dental erosion.

 Additionally, the SEM analysis allowed for confirmation of the outcomes. The structural disintegration and enamel surface destruction seen in SEM micrographs correlated with the tooth samples’ weight loss percentages. The only tooth samples that showed no erosion were those immersed in the Alive! Kids multivitamin, and this was consistent with the fact that the weights of the tooth samples remained constant.

 The researchers in this experiment tried to simulate the environment of the oral cavity and the consumption of daily vitamin supplements by immersing tooth samples in vitamin solutions and artificial saliva in a periodic cycle at 37 °C. The tooth samples were exposed to vitamin supplements twice a day, as recommended on the label of the chewable vitamin products, which suggested taking one tablet in the morning and another in the evening. Tooth samples were immersed in a vitamin solution for 1 hour based on the food elimination and retention times on the tooth surfaces.^21^

 This study had some limitations. Because it was an in vitro investigation, it could not simulate the situation in life during which vitamin tablets are chewed and crushed by the occlusal surface of a tooth; instead, chewable vitamin tablets were crushed and dissolved in solutions. It also could not simulate how salivary flow in the oral cavity affects the rate at which food is eliminated or the saliva’s buffering capacity.^22^ Nevertheless, the findings of this study demonstrated that vitamin supplements in the form of chewable tablets did have an impact on the dissolution and loss of tooth enamel and could cause dental erosion.

 Nowadays, because vitamin supplements are promoted as nutritional products that provide health benefits, they have become popular with children.^23^ Since some parents and caregivers are unaware of the harmful impacts of vitamin supplements on tooth mineral structures, they would rather give their children chewable vitamin tablets than candy or other sweets. They believe vitamins can strengthen the immune system and are especially attuned to this in light of the COVID-19 pandemic.^24^ According to this study, most chewable vitamin supplements tested could erode enamel and cause the loss of the mineral components and structure of teeth. All the tested supplements were acidic. However, the erosive effects were less pronounced in multivitamins containing mineral calcium.

## Conclusion

 All of the children’s chewable vitamin supplements tested in this study were acidic, with lower pH values than enamel’s critical pH. These supplements could potentially erode the enamel surface, leading to the loss of the mineral structure of teeth. Chewable vitamin C supplements tended to have a lower pH than chewable multivitamin supplements, and the lower the pH, the greater the erosion of the enamel surface. The erosive potential was less in multivitamins containing calcium as part of their mineral content. Healthcare providers and caregivers should be aware of the potential harms of chewable vitamin supplements on teeth.

## Competing Interests

 Authors have no conflict of interest to declare.

## Ethical Approval

 The ethics of this study was approved by the Institutional Review Board (COE.No.MU-DT/PY-IRB 2023/055.2811) of the Faculty of Dentistry/Faculty of Pharmacy, Mahidol University.

## Supplementary Files

Supplementary file 1 contains table S1.
