# Supplementary material for: Erosive potential of children’s chewable vitamin supplements: An in vitro investigation
Source: J Dent Res Dent Clin Dent Prospects. 2024 Dec 14;18(4):278–83. doi: 10.34172/joddd.41791 (PMC11786007; doi:10.34172/joddd.41791)
Supplement: Supplementary file 1 — contains table S1. [file joddd-18-278-s001.pdf]

Lertsooksawat et al, *J Dent Res Dent Clin Dent Prospects,* 2024, 18(4), 278-S1
doi: 10.34172/joddd.41791
https://joddd.tbzmed.ac.ir

Supplementary file 1

Table S1. Children’s chewable vitamin supplements used in this study

Groups and products	Manufacturers	Major ingredients
Vitamin C		
Vita-C orange flavor	Heaven Herb Co., Ltd., Pathum Thani, Thailand	Vitamin C, Sugar, Flavoring agent
NaturesPlus Vitamin C Children’s Chewable Supplement	Natural Organic, Inc. New York, USA	Vitamin C, Xylitol, Bioflavonoids, Natural flavors, Natural color, Stearic acid, magnesium stearate, C-source, Bilberry, Papaya, Guava, Pineapple and Silica
BigFriends Chewable Vitamin C	Natural Factors Canada., Monroe, USA	Vitamin C, Xylitol, Sodium ascorbate, Citrus Bioflavonoids Extract, Hesperidin Extract, Rosehips
Multivitamin		
21st Century Children’s Multivitamin Supplement	21st Century HealthCare, Inc. Arizona, USA	Vitamin A, Vitamin C, Vitamin D3, Vitamin E, Thiamin, Niacin, Riboflavin, Vitamin B6, Folate, Vitamin B12, Sodium, other ingredients
NaturesPlus Multivitamin Children’s Chewable Supplement	Natural Organic, Inc. New York, USA	Sugars, Vitamin A, Vitamin C, Vitamin D, Vitamin E, Thiamin, Riboflavin, Niacin, Vitamin B6, Folate, Vitamin B12, Biotin, Pantothenic acid, Calcium, Iron, Magnesium, Zinc, Copper, Pineapple fruit, Apple fruit, Sunflower seed oil, Lemon bioflavonoid complex, PABA, Inositol
Alive! Kids Chewable Multivitamin	Nature's Way Brands, LLC, Wisconsin, USA	Vitamin A, Vitamin C, Vitamin D3, Vitamin E, Thiamin, Niacin, Riboflavin, Vitamin B6, Folate, Vitamin B12, Biotin, Pantothenic Acid, Choline, Calcium, Iron, Iodine, Magnesium,

		Zinc, Manganese, Molybdenum, Orchard Fruits and Garden Veggies, Citrus bioflavonoid complex
